# Revolutionizing healthcare: the transformative potential of nanotechnology in medicine

**DOI:** 10.3389/fddev.2025.1556426

**Published:** 2025-05-30

**Authors:** Lavan K. Durgam, Terry L. Oroszi

**Affiliations:** Department of Pharmacology and Toxicology, Boonshoft School of Medicine, Wright State University, Fairborn, OH, United States

**Keywords:** nanomedicine, drug delivery, tissue engineering, nanomaterial-based vaccines, artificial intelligence in nanotechnology

## Abstract

Medical science stands on the brink of transformation thanks to nanotechnology’s fast-paced development, which promises major advancements. Working with materials at the nanoscale within a 1–100 nm range allows scientists to tap into special physicochemical characteristics that open up new possibilities in diagnostics, drug delivery, and regenerative medicine. The review explores nanotechnology’s revolutionary effects on healthcare by highlighting its roles in medical imaging applications and diagnostic procedures, drug delivery systems, tissue engineering, and vaccine development. The design principles of nanomaterials, which encompass synthesis methods alongside functionalization and characterization techniques, are presented here. This review analyzes the impact of artificial intelligence in nanomedicine alongside the enduring effects of nanomaterials and related ethical and safety issues. The review further combines multiple study findings to offer a thorough overview of nanotechnology’s medical applications while suggesting research and clinical translation paths.

## 1 Introduction: nanotechnology in medicine

The rapid advancement of nanotechnology offers significant potential to revolutionize medical practices (Sahu et al., 2023). Nanoscale material manipulation between 1 and 100 nm allows access to unique physicochemical properties that bulk materials cannot exhibit. The ongoing developments in nanotechnology research generate new opportunities for diagnostic tools, drug delivery systems, and advances in regenerative medicine (Chavda, 2019; Malik et al., 2023).

Nanotechnology in medicine improves biocompatibility while developing systems for targeted delivery that can overcome biological barriers. A fundamental examination of both biocompatibility and nanotoxicology is required to ensure the clinical safety of these materials. Sharma et al. (2024) investigates current and anticipated clinical research applications of nanotechnology and highlights its revolutionary potential in healthcare advancement. Additionally, Sahoo et al. (2017) analyzed existing and potential nanotechnology applications in healthcare and highlighted its ground-breaking capabilities for medical practice.

The study examines the medical applications of nanotechnology in diagnostics and drug delivery systems, as well as regenerative medicine, and highlights technical and ethical issues.

**FIGURE 1:**
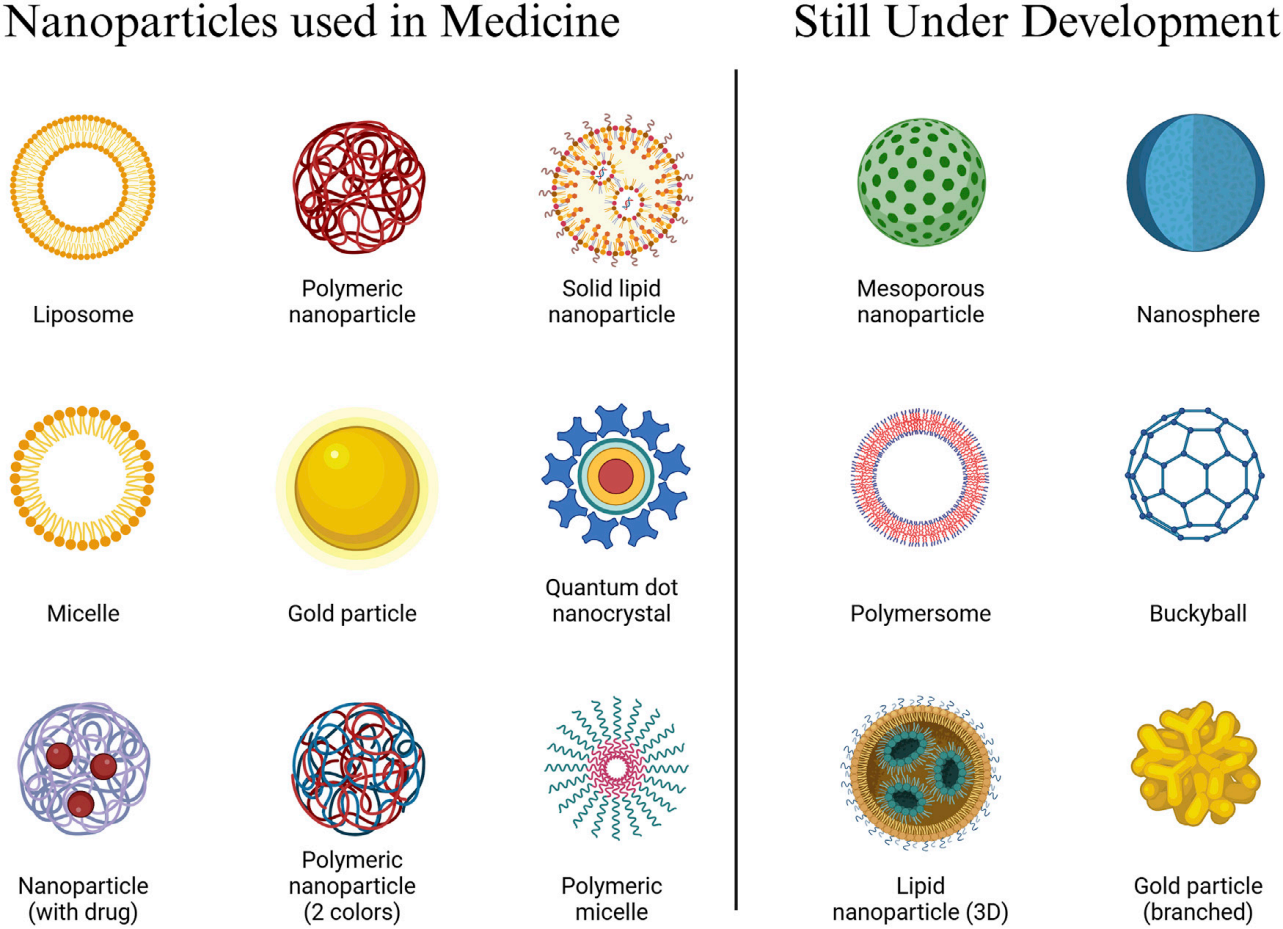
Nanoparticles Used in Medicine and Under Development. The field of nanomedicine demonstrates substantial abilities to improve therapeutic outcomes while reducing harmful side effects. These materials operate at the nanoscale level to transport therapies directly into certain cells or tissues. According to research by Chavda (2019), liposomes help deliver drugs while decreasing the toxicity effects on healthy tissues. The design of polymeric nanoparticles enables them to penetrate the blood-brain barrier for precise delivery, as demonstrated by Chen et al. (2023). Gold particles improve both resolution and specificity for imaging, as shown by Singh and Deshmukh, (2023). Clinical trials confirmed the high effectiveness of nanomaterial-based vaccines against COVID-19, based on research by Wilson and Geetha in 2022.

Nanoparticles are currently used in medical applications and there are several still under development (See Figure 1). The diagram displays many nanomaterials used in medical applications, including liposomes, polymeric nanoparticles, solid lipid nanoparticles, gold particles, and quantum dots. Nanotechnology enables healthcare transformation by enabling precise drug delivery systems, advanced imaging capabilities, and innovative treatment methods.

### 1.1 Overview and advantages

Nanomedicine exhibits powerful capabilities for increasing treatment effectiveness and minimizing adverse side effects. These materials work at the nanoscale to deliver treatments directly to specific cells or tissues. Nanoparticle engineering provides targeted drug delivery to cancer cells while reducing harm to normal tissues (Chavda, 2019; Occhiutto et al., 2020). Nanomaterials improve imaging technologies to produce detailed and clearer images that facilitate early disease detection and monitoring of disease (Chavda, 2019; Malik et al., 2023).

### 1.2 Biocompatibility and nanotoxicology

Relies heavily on the ensured biocompatibility of the materials used. To achieve safe biological interactions, nanomaterials must be carefully designed to avoid triggering adverse immune responses. Rigorous testing procedures must be implemented to ensure that nanomaterial interactions with cells and tissues maintain safety and avoid adverse immune responses. Nanotoxicology research helps assess nanomaterial risks since they can cause oxidative stress and inflammation alongside cytotoxic reactions (Tekade et al., 2017; Pelaz et al., 2017).

### 1.3 Limitations and challenges

Several obstacles need resolution before medical nanotechnology can reach its potential for safe and effective application. Incomplete knowledge about the long-term impacts of nanomaterials on human health and environmental systems has led to concerns about their potential effects (Schneider-Futschik and Reyes-Ortega, 2021). Research must persistently explore nanomaterials’ full safety profile while developing strategies to minimize their potential risks. Nanomaterials offer clear benefits for drug delivery and imaging applications but also show toxic effects and cause adverse immune responses (Liu et al., 2022). EL-Khawaga et al. (2023) described how the complex and costly production processes of nanomaterials act as barriers to their large-scale production. Standardized protocols must evolve within regulatory frameworks to ensure nanomedicine safety and effectiveness during approval (Tekade et al., 2017; Pelaz et al., 2017).

Although nanomaterials offer targeted drug delivery capabilities, scientists face significant challenges when crossing biological barriers such as the blood-brain and reticuloendothelial systems. Precise delivery of drugs to their designated sites while avoiding side effects in other areas must be achieved to ensure therapeutic success (Chen et al., 2023). Developing optimized drug delivery systems and better nanomedicine efficacy requires additional research. Moving laboratory nanotechnology research into clinical practice faces substantial hurdles related to safety regulations and manufacturing hurdles. The complexity of biological systems combined with the variability of patient response creates substantial obstacles to executing clinical trials and the broad adoption of nanomedicine technologies.

## 2 Design principles of nanomaterials

### 2.1 Nanomaterial definition and production

Materials qualify as nanomaterials with one dimension between 1 and 100 nm. According to the European Commission, nanomaterials include substances with particles that measure between 1 and 100 nm for half their external diameters, whether those particles are natural or synthesized. To qualify as a nanomaterial, a structure must measure between 1 and 100 nm in at least one exterior dimension. Fullerenes, graphene flakes, and single-walled carbon nanotubes belong to this category. Any material with a surface area to volume ratio above 60 m^2^/cm^3^ falls within the included category. The FDA lacks formal definitions for terminology such as “nanotechnology,” “nanomaterial,” and “nanoscale,” unlike the standard practice seen when engineering materials with dimensions between 1 and 100 nm (nm). According to the FDA, evaluating nanotechnology-derived products for efficacy, safety, public health impact, or regulatory status should not involve component isolation because information on nanoparticles and their characteristics is insufficient. The 2020 study by Singh and Deshmukh (2023) provides a concise introduction to nanomaterials, emphasizing their properties and highlighting their widespread applications across different fields.

### 2.2 Synthesis methods and process control

Bottom-up and top-down approaches serve as production methods for nanomaterials within the pharmaceutical industry. The top-down process involves breaking down large materials into small pieces by applying mechanical or chemical energy. In contrast to top-down methods, the bottom-up approach builds materials by enlarging small molecular or atomic units through chemical reactions. Both manufacturing processes generate primary particles along with aggregates and agglomerates. An agglomerate represents a loose collection of particles or aggregates whose combined external surface area matches the total surface areas of all individual components. An aggregate forms when individual particles bind together strongly or fuse into a single unit. A particle represents a small segment of matter characterized by precise physical limits. The definition incorporates aggregates and agglomerates that retain their unbound characteristics after breaking down into nanoscale components.

The production of nanomaterials involves two main synthesis methods: top-down and bottom-up. The top-down approach generates nanoscale particles by breaking down larger materials using mechanical or chemical energy, while the bottom-up approach constructs nanomaterials by assembling smaller atomic or molecular entities through chemical reactions (Khan et al., 2015; Abidin et al., 2024). These methods produce primary particles, aggregates, and agglomerates with specific properties and applications (Lövestam et al., 2010). The top-down approach utilizes milling, lithography, and etching techniques to produce nanomaterials of precise dimensions, though it carries the risk of defects and impurities (Abidin et al., 2024). Conversely, the bottom-up approach employs chemical vapor deposition, sol-gel processes, and self-assembly methods to produce nanomaterials of high purity with controlled composition and structure (Khan et al., 2015).

The pharmaceutical production process requires rigorous oversight that demands the determination of key factors and the creation of suitable evaluation instruments. Quality-by-Design (QbD) is dependent on process analytical technologies (PATs) to evaluate and regulate nanomedicines systematically (Aguiam et al., 2025). The PAT system enables manufacturers to monitor and control production processes in real-time to maintain consistent quality and performance standards for nanomedicines (Aguiam et al., 2025). The described approach enables the detection of both critical quality attributes (CQAs) and critical process parameters (CPPs) that affect the final product quality (Gaspar, 2010).

The production of medical nanomaterials requires multiple essential procedures, starting with carefully selecting raw materials to maintain purity and quality. Synthesis constitutes the subsequent stage in which top-down or bottom-up approaches generate nanomaterials. Functionalization occurs next, including surface modifications to improve properties and biological system interactions (Ahmad et al., 2022). Assessment of physicochemical characteristics, including size, shape surface area, and chemical composition, relies heavily upon characterization methods (Soares et al., 2018). The formulation stage involves the integration of nanomaterials into drug delivery systems and other medical applications. The quality control mechanism uses process analytical technologies (PAT) to achieve real-time monitoring and control during manufacturing processes. The stability and integrity of nanomaterials during transportation and storage depend on effective packaging and storage procedures.

**FIGURE 2:**
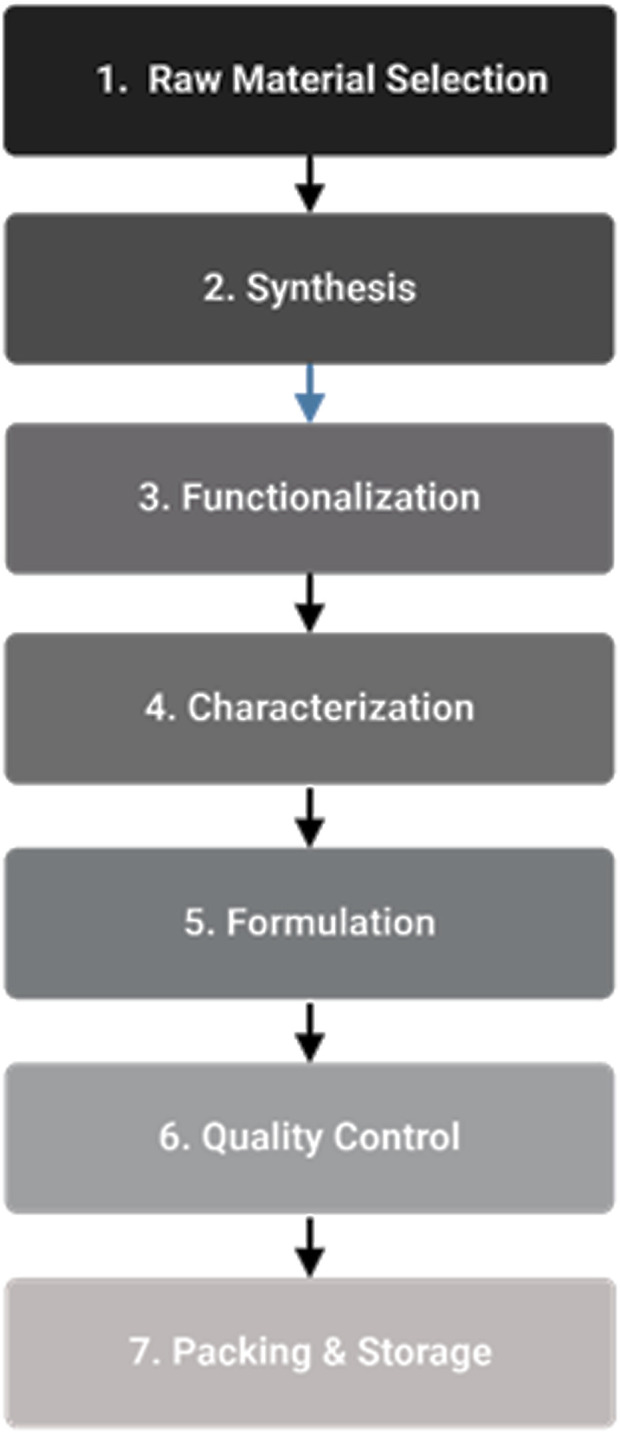
Flow chart of critical manufacturing steps.

The synthesis of nanomaterials involves top-down methods for precise size control (Abidin et al., 2024). Characterization techniques assess the physicochemical properties of nanomaterials, including size, shape, surface area, and composition (Soares et al., 2018). Quality control processes employ Process Analytical Technologies (PAT) to ensure consistent quality and performance of nanomedicines (Aguiam et al., 2025). See Figure 2 for the steps in critical manufacturing.

Flow Chart of Critical Manufacturing Steps:

1. Raw Material Selection: Ensuring the purity and quality of raw materials.
2. Synthesis: Employing top-down or bottom-up approaches to create nanomaterials.
3. Functionalization: Modifying the surface of nanomaterials to enhance their properties and interactions with biological systems (Ahmad et al., 2022).
4. Characterization: Assessing the physicochemical properties of nanomaterials, including size, shape, surface area, and chemical composition (Soares et al., 2018).
5. Formulation: Incorporation of nanomaterials into drug delivery systems or other medical applications.
6. Quality Control: Implementing PAT to monitor and control the manufacturing process in real-time.
7. Packaging and Storage: Ensuring the stability and integrity of nanomaterials during storage and transportation.

### 2.3 Functionalization

Surface modification through functionalization enhances the biological interaction properties of nanomaterials (Ahmad et al., 2022). Effective targeting of specific biological processes requires modifications to the surface chemistry, size, shape, and composition of materials. Functionalization plays a critical role in achieving specific biological targets, such as directed drug delivery and improved diagnostic precision (Fleischer and Payne, 2014). When polyethylene glycol (PEG) is added to nanomaterials, it enhances both biocompatibility and targeting capabilities (Shi et al., 2021). PEGylation protects nanomaterials from immune system detection, extending their presence in the bloodstream and improving delivery to target tissues (Guo et al., 2023). This technique is frequently used in drug delivery systems to increase safety and effectiveness (Tekade et al., 2017; Guo et al., 2023).

## 3 Characterization of nanomaterials

The evolution of nanomaterials for medical applications demands thorough characterization as a vital step. The procedure involves identifying various properties that influence nanomaterial behavior, such as size, shape, surface area, and chemical composition (Soares et al., 2018). The properties of nanomaterials determine their biological interactions and their safety and performance.

### 3.1 Characterization techniques and their importance

Using various techniques, researchers determine the specific properties of nanomaterials. Table 1 provides a list of primary characterization methods together with the corresponding parameters they measure. Characterization is essential for several reasons:

**TABLE 1:** Key characterization techniques and the parameters measured.

Technique	Parameter	Description	References
Transmission Electron Microscopy (TEM)	Size, Shape	Provides high-resolution images to determine the size and shape of nanoparticles	Chaturvedi et al. (2019)
Scanning Electron Microscopy (SEM)	Surface Morphology	The detailed images of the surface structure and morphology of nanomaterials	Chaturvedi et al. (2019)
Dynamic Light Scattering (DLS)	Size Distribution	Measures the size distribution of the nanoparticles in suspension	Farkas and Kramar, 2021
X-ray Diffraction (XRD)	Crystal Structure	Determines the crystalline structure and phase composition of nanomaterials	Chaturvedi et al. (2019)
Brunauer-Emmett-Teller (BET) Analysis	Specific Surface Area	Measures the surface area of nanomaterials, which is crucial for understanding their reactivity	Nagarajan (2008)
Fourier Transform Infrared Spectroscopy (FTIR)	Chemical Composition	Identifies the chemical bonds and functional groups present on the surface of nanomaterials	Chaturvedi et al. (2019)
Zeta Potential Analysis	Surface Charge	Measures the surface charge of nanoparticles, which affects their stability and interactions	Anjum et al. (2021)
Thermogravimetric Analysis (TGA)	Thermal Stability	Assesses nanomaterials’ thermal stability and composition by measuring weight changes upon heating	Chaturvedi et al. (2019)
Inductively Coupled Plasma Mass Spectrometry (ICP-MS)	Elemental Composition	Provides a quantitative analysis of the elemental composition of nanomaterials	Chaturvedi et al. (2019)
Ultraviolet-visible spectroscopy (UV-Vis)	Optical Properties	Measures the absorbance and transmittance of nanomaterials to determine their optical properties.	Chaturvedi et al. (2019)

#### 3.1.1 Safety evaluation

Assessing potential nanomaterial toxicity and biological system interactions depends on understanding their physicochemical properties (Soares et al., 2018).

#### 3.1.2 Process control

The nanomaterial manufacturing process uses characterization techniques to maintain consistent quality and performance (Aguiam et al., 2025).

#### 3.1.3 Regulatory compliance

To obtain regulatory approval for nanomedicines, researchers must perform a comprehensive characterization as dictated by regulatory standards and guidelines (Tinkle et al., 2014).

#### 3.1.4 Optimization of properties

Through nanomaterial characterization, researchers achieve optimization of their properties for specific uses such as drug delivery or imaging (Chaturvedi et al., 2019; Narayanaswamy et al., 2016).

The complete understanding of nanomaterials necessitates the use of multiple characterization techniques. Researchers can simultaneously employ TEM and SEM to examine nanoparticles’ internal structure and surface morphology. Using DLS and zeta potential analysis together shares information about nanoparticle size distribution and suspension stability.

## 4 Applications of nanotechnology

The medical field is experiencing a revolution thanks to multiple innovative nanotechnology applications. Nanoscale material manipulation enables researchers to exploit special physicochemical attributes to develop targeted drug delivery systems, advanced imaging, and new therapeutic strategies. These technological breakthroughs substantially enhance patient recovery results for multiple disease types.

**FIGURE 3:**
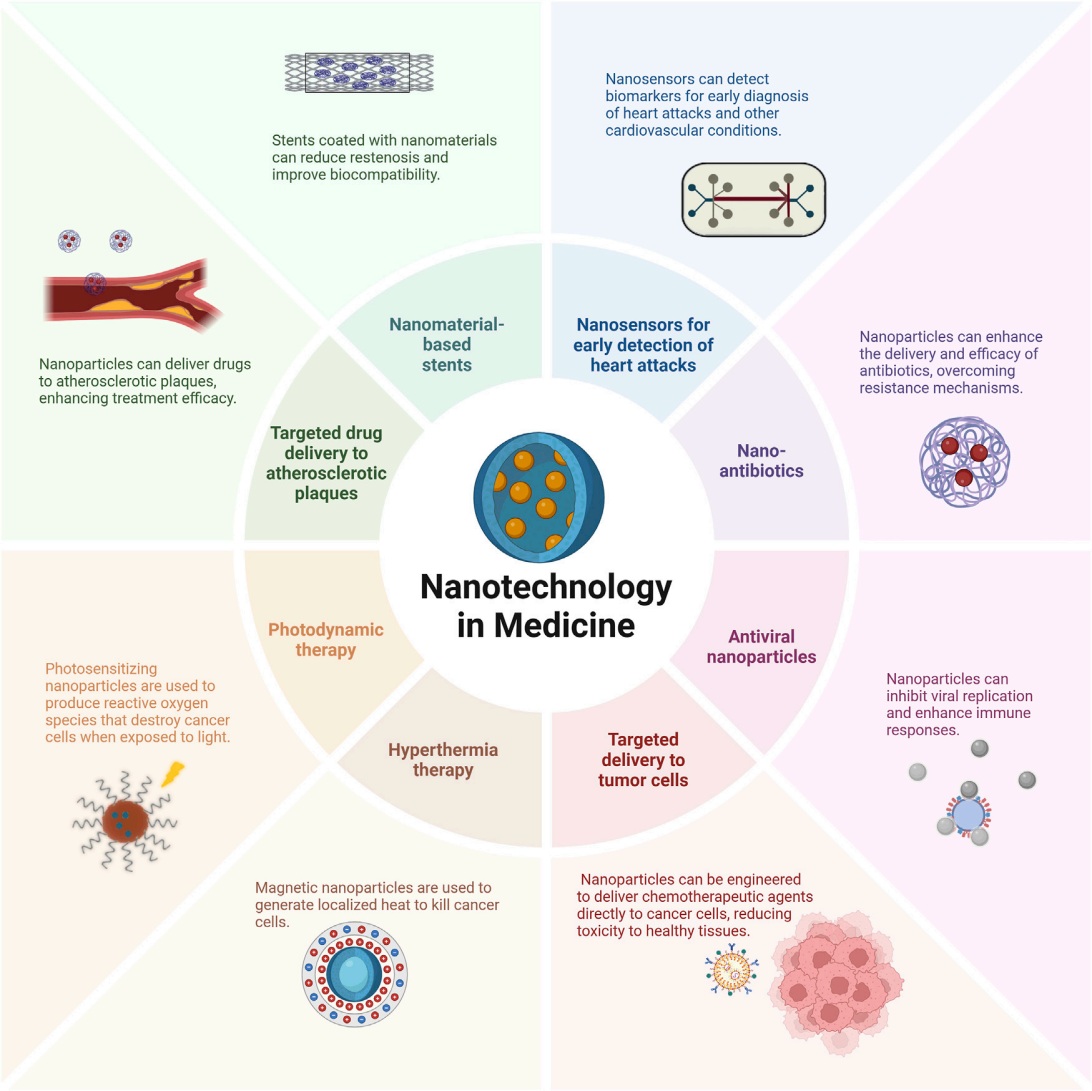
Diverse Applications of Nanotechnology in Medicine. Nanotechnology has multiple medical applications, including drug delivery to tumor cells and atherosclerotic plaques, magnetic nanoparticles for hyperthermia therapy, photosensitizing nanoparticles for photodynamic therapy, and nano-antibiotics to combat infectious diseases. This visual representation highlights nanotechnology’s potential to transform healthcare by providing specific and efficient treatments for various diseases. Targeted drug delivery to tumor cells reduces toxicity to healthy tissues (Chavda, 2019; Naseri et al., 2018). Nanosensors offer improved sensitivity for early heart attack detection (Elsaygh et al., 2024). Nanomaterial-based stents reduce restenosis and improve biocompatibility (Rizvi and Saleh, 2018).

Nanotechnology finds multiple medical applications, including delivering drugs to tumor cells and atherosclerotic plaques, using magnetic nanoparticles in hyperthermia therapy, photosensitizing nanoparticles in photodynamic therapy, and nano-antibiotics to combat infectious diseases. Through visual representation in Figure 3, it becomes clear that nanotechnology has the potential to transform healthcare by delivering specific and efficient treatments for various diseases.

### 4.1 Nanotechnology in medicine

#### 4.1.1 Cancer

Nanotechnology is transforming modern medical practice through multiple breakthrough applications including targeted drug delivery systems, hyperthermia therapy approaches, and photodynamic therapy (PDT) (Alexis et al., 2008; Sher et al., 2024). Nanomaterials deliver chemotherapeutic drugs directly to cancerous cells while protecting healthy tissues from damage (Chavda, 2019; Naseri et al., 2018). Doxil for doxorubicin and Abraxane for paclitaxel represent examples of liposomal formulations that function as targeted delivery systems to decrease toxicity in healthy tissues (Chavda, 2019; Naseri et al., 2018).

Nanomaterials that respond to magnetic fields produce localized heat to kill cancer cells in hyperthermia therapy, whereas nanomaterials that photosensitize create reactive oxygen species to destroy cancer cells in photodynamic therapy. Research by Contera et al. highlights the potential of hydrogels to deliver therapeutic agents with control in breast cancer treatments. (2020), Nel et al. (2023), and Buchheit et al. (2021).

The development of advanced cancer treatment methods, such as photodynamic therapy (PDT) and immunotherapy, is heavily dependent on nanotechnology. Research by Lakkakula et al. (2024) and Jia et al. Research by Jia et al. Jia et al. (2023) study reveals that nanotechnology improves photosensitizer delivery performance and safety while increasing immunotherapy agent effectiveness. The combination of PDT and immunotherapy exceeds the limits of individual therapies through enhanced antitumor activity. The design of nanoparticles improves the solubility and stability of photosensitizers, allowing for controlled distribution and release. Targeting ligands can be attached to nanoparticles to improve tumor targeting and offer photosensitizers that work for imaging and therapy.

Cancer treatment through nanotechnology revolutionized oncology by offering exact treatments targeting cancer cells while maintaining minimal invasiveness (Sayyad et al., 2025). Nanoparticles possess unique physicochemical attributes that enable them to function as multifunctional agents that improve drug delivery systems with imaging and therapeutic results.

##### 4.1.1.1 Cancer case studies

###### 4.1.1.1.1 Case Study 1

Lakkakula and team (2024) analyzed emerging advancements in cyclodextrin (CD)-based nanoparticles used for cancer treatment through photodynamic therapy. The authors presented a complete review of scientific studies examining the applications of CD nanoparticles in advancing drug delivery systems and photodynamic therapy outcomes. CD-based nanoparticles demonstrate improved light absorption and drug-loading abilities, facilitating hydrophobic photosensitizers’ controlled delivery and release within cancer cells.

###### 4.1.1.1.2 Case Study 2

Pareek et al. (2025) examined advances in drug delivery systems by deploying quantum dots (QDs) and various nanostructures. The research analyzes several chemical linking techniques for QDs, including covalent binding and noncovalent conjugation, click chemistry, and disulfide- and pH-sensitive linkages. QDs serve as drug delivery tools across various cancer treatments while offering real-time imaging capabilities and improved therapeutic outcomes. The research team explores multiple challenges related to QDs, such as their potential toxicity and stability, while highlighting issues with pharmacokinetics and target accuracy.

#### 4.1.2 Cardiovascular disease

The treatment of heart disease benefits from nanotechnology through targeted drug delivery systems combined with nanomaterial-coated stents and early detection capabilities of nanosensors. Nanomaterials facilitate the administration of targeted drugs directly to atherosclerotic plaques, which enhances treatment results (Chen et al., 2021). HA-ATV-NP demonstrate superior anti-inflammatory effects because they bind to CD44 receptors that are excessively present on cells at atherosclerotic plaque sites compared to free atorvastatin (Elsaygh et al., 2024).

Stents coated with nanomaterials achieve reduced restenosis rates while simultaneously enhancing biocompatibility. Nanosensors enable early detection of cardiovascular conditions by identifying biomarkers for heart attacks through studies by Rizvi and Saleh (2018), Tekade et al. (2017), Occhiutto et al. (2020) and Perera et al. (2023). These biosensor nanomaterials utilize components such as gold nanoparticles and zinc stannate to enhance molecule binding specificity and reduce nonspecific adsorption, thus boosting detection sensitivity (Elsaygh et al., 2024). Scientists work to create gold and silica nanoparticles to enhance the distribution of nitric oxide for vascular health (Golchin et al., 2018).

Lemos et al. (2024) developed a specialized nanosystem that produces oxygen to treat infarcted heart muscle cells by activating ultrasound while protecting nearby tissues from damage after a heart attack. The field of cardiac tissue engineering utilizes nanotechnology by investigating nanocomposite polymeric materials and hydrogel-based scaffold structures that demonstrate capabilities to regenerate cardiac tissue and improve cardiomyocyte functionality (Elsaygh et al., 2024).

##### 4.1.2.1 Cardiovascular disease case studies

###### 4.1.2.1.1 Case Study 1

The work of Lemos et al. (2024) showed several nanotechnology applications for cardiovascular therapy that demonstrated minimal side effects and high effectiveness for CVD prevention and treatment. Research found that several triggers, including internal pH, hypoxia levels, external temperature, and light exposure, can trigger nanocarriers to release drugs.

###### 4.1.2.1.2 Case Study 2

Elsaygh et al. (2024) investigated how nanotechnology enables rapid and accurate diagnostic methods for the early detection of coronary artery disease. The study examines nanoparticles designed for accurate imaging applications that use 18F PET imaging to detect inflammation and macrophage activity. The research identifies different categories and their specific functions while pointing out that lipid-based nanoparticles enable targeted drug administration and polymeric nanoparticles perform drug delivery tasks.

#### 4.1.3 Infectious disease

Nanotechnology provides key solutions for controlling infectious diseases by creating nanoantibiotics, antiviral nanomaterials, and vaccine technologies (Huang et al., 2024). The study by Chen et al. (2021) shows that nanomaterials improve antibiotic delivery effectiveness while breaking down resistance mechanisms. (2021). The successful laboratory research on the antibacterial and antiviral properties of AgNPs, selenium nanoparticles (SeNPs), and metal oxide NPs shows their suitability for environmental sanitation and therapeutic or preventive inhalation applications.

Antiviral nanomaterials prevent viral replication while enhancing the immune system’s functions. Research shows that vaccines utilizing nanomaterials deliver better performance and stability (Chaturvedi et al., 2019; Pelaz et al., 2017; Chen et al., 2021). Nanotechnology boosts antimicrobial vaccines Through enhanced biocompatibility, immunogenicity, and superior antigen presentation abilities. Lipid nanoparticles, polymeric nanoparticles, and exosomes function as drug delivery)systems to facilitate precise targeting and controlled drug release, enhancing treatment effectiveness.

##### 4.1.3.1 Infectious disease case studies

###### 4.1.3.1.1 Case #1

Nanotechnology in the COVID-19 pandemic. The appearance of the SARS-CoV-2 virus triggered the COVID-19 pandemic, which resulted in a worldwide health emergency. Scientific researchers, including Yang (2022), have concentrated on creating effective vaccines and treatments to combat the virus. Nanotechnology stands out as a viable approach to solving worldwide health issues. Nanoparticle-based delivery systems have enhanced intranasal drug administration by overcoming mucosal administration barriers and increasing delivery effectiveness.

Nanotechnology tools allow researchers to create advanced vaccine design strategies and improve therapeutic delivery techniques. Lipid nanoparticles support antiviral molecule delivery through intranasal routes, and polymer nanoparticles enhance drug penetration and effectiveness. Nanotechnology platforms enable developers to rapidly detect SARS-CoV-2 alongside cost-effective testing, aiding vaccine development and immunoengineering research. Progress in nanotechnology demonstrates its potential to develop innovative approaches against infectious diseases.

###### 4.1.3.1.2 Case #2

Nanotechnology in the COVID-19 pandemic. The COVID-19 pandemic emerged when the SARS-CoV-2 virus instigated a global health crisis. Researchers like Yang (2021) have spent their careers developing effective vaccines and treatments to combat the virus. Nanotechnology has significant potential as a solution to this critical problem. Developing intranasal delivery systems through nanoparticle-based drug delivery methods resolves mucosal administration difficulties and improves overall drug delivery performance.

Nanotechnology tools enable revolutionary advances in how vaccines are designed and the delivery of therapeutic systems. Lipid nanoparticles facilitate antiviral molecule delivery through intranasal administration, and polymer nanoparticles improve drug penetration and effectiveness. Developers are constructing nanotechnology platforms that facilitate rapid and economical detection of SARS-CoV-2 and help with vaccine research and immunoengineering efforts. Advances in nanotechnology reveal its potential to create innovative strategies to fight infectious diseases.

#### 4.1.4 Neurodegenerative diseases

The intersection of precise drug delivery techniques with neural regeneration capabilities and nanosensor detection systems provides nanotechnology with multiple effective treatment approaches for neurodegenerative diseases (He et al., 2022). Nanomaterials can cross the blood-brain barrier (BBB), which allows the direct administration of medication for diseases such as Alzheimer’s and Parkinson’s. Scientists in lipid-based, polymeric, metallic, and carbon-based nanoparticle research efforts aim to create new disease treatments. Nanosensors provide researchers with the ability to identify early disease markers, which enhances both diagnostic methods and treatment approaches for neurodegenerative conditions. He et al. demonstrated that nanomaterials facilitate neural tissue repair and growth, which holds therapeutic benefits for neuronal damage repair. (He et al., 2022).

Nanotherapeutics show potential for treating neurodegenerative diseases through improved drug delivery through the blood-brain and blood-cerebrospinal fluid barriers (B- CSFB). Scientists deploy nanoparticles to tackle detection and therapeutic challenges in Alzheimer’s, Parkinson’s, and Huntington’s diseases. Nanomedicine innovations are essential to address complex disorders because they must improve drug solubility and circulation time while delivering medications to specific targets and avoiding unwanted effects (Dhariwal et al., 2025). The potential of nanotechnology in treating Parkinson’s disease exists because it addresses the limitations of traditional therapy. Nanomedicine uses nanoparticles, which capitalize on their diminutive size and substantial surface area-to- volume ratio to cross the BBB and deliver drugs straight to brain areas affected by the disease. Research shows that lipid-based nanoparticles, polymeric and metallic nanoparticles, and carbon-based nanoparticles exhibit potential as therapies for Parkinson’s disease. Systems using nanocarriers, including liposomes and solid lipid nanoparticles, enable the precise release of therapeutic agents that improve bioavailability and reduce side effects (Yadav et al., 2025).

Nanotechnology shows the potential to address existing medical limitations and create new approaches to treating neurodegenerative diseases. Nanoparticles have shown the ability to cross the BBB, which could lead to innovative diagnostic and treatment methods for various brain disorders. Combining nanotechnology with gene therapy and nanomaterial-based research methods opens new opportunities for enhancing treatment outcomes and stem cell differentiation applications in neural cell therapies.

##### 4.1.4.1 Neurodegenerative disease case studies

###### 4.1.4.1.1 Study #1

A study by Singh et al. (2022) examined Huntington’s disease, which stems from a genetic mutation in the huntingtin gene. The accumulation of mutant huntingtin protein within brain cells causes the disease to manifest through motor impairments and cognitive and psychiatric symptoms. Nanotechnology is a promising delivery method that allows therapeutic agents to reach the brain and maintain stability. Polymeric nanoparticles serve as transport vehicles for drugs to cross the blood-brain barrier. Researchers have developed polymeric nanoparticles that carry a peptide-based polyglutamate aggregation inhibitor to specific cells.

Applying these nanoparticles in cellular and *Drosophila* HD models successfully reduced protein aggregation while maintaining biocompatibility. Research shows how nanotechnology-based drug delivery systems offer promising treatment options for Huntington’s disease by directly targeting its molecular root causes.

#### 4.1.5 Orthopedics

Nanomaterials are revolutionizing orthopedics by supporting bone tissue engineering and producing nanocomposites for bone implants. Orthopedics has made significant progress through the implementation of nanotechnology-based drug delivery systems. Nanobased scaffolds and nanoparticles are common treatments for bone-related conditions such as osteoarthritis, osteosarcoma, cancer bone metastasis, osteoporosis, bone infections, and inflammatory diseases (Chaturvedi et al., 2019; Tekade et al., 2017). The repair and regeneration processes of bone tissue depend heavily on their function.

Antibacterial nanomaterials demonstrate significant promise for *preventing bone infections* because they increase antibiotic performance and breakthrough bacterial defenses. Antibiotic delivery systems based on nanotechnology provide multiple benefits compared to conventional antibiotics, including sustained drug release control and surface modification capabilities for specific targeting of bacteria or bone tissue, along with high local bioactivity and minimal systemic side effects, and can deliver multiple antibiotics while improving drug solubility and stability (Güven, 2021).

Ruan and his team (2024) reviewed recent advancements in orthopedic nanotechnology while investigating new nanofiber applications for tendon repair and studying selenium and cerium oxide nanoparticles for osteoarthritis treatment and osteoblast differentiation. This paper discusses new injectable hydrogel applications for cartilage engineering, which requires interdisciplinary research, and points out current difficulties and future opportunities for integrating nanotechnology into orthopedic treatments. The applications in bone regeneration establish innovative methods to treat various orthopedic diseases. Versatile and efficient nanotechnology merges various orthopedic treatment approaches, making it an essential research field for future medical applications and clinical practice.

Nanomaterials offer promising applications in orthopedic graft fabrication because they can replicate natural bone components (Wang et al., 2016; Cheng et al., 2018). Orthopedic management depends on bone replacement procedures to repair damage that cannot be repaired in healthy native bone. Nanomaterials provide essential cellular scaffold support through nanofunctionalization, affecting how cells propagate and differentiate during migration (Patel et al., 2016; Vieira et al., 2017).

##### 4.1.5.1 Orthopedic case studies

###### 4.1.5.1.1 Case Study #1

Ruan et al. (2024) investigated how nanotechnology could revolutionize orthopedic treatments. The researchers examined current advancements in orthopedic nanotechnology while investigating how nanoscale materials can be applied to treating orthopedic conditions. The study revealed nanofiber scaffolds to repair tendons alongside selenium and cerium oxide nanoparticles for the treatment of osteoarthritis and injectable hydrogels as tools for cartilage engineering. Research confirmed that nanotechnology can improve the treatment approaches for orthopedic diseases.

###### 4.1.5.1.2 Case Study #2

Chen’s 2022 work explored how biomaterials for orthopedic applications have evolved from traditional substances to nanoparticles and how they function as drug delivery platforms. The study aimed to examine recent developments in orthopedic biomaterials by focusing on smart biomaterials and porous materials while exploring 3D-printable nanocomposite grafts. Nanomaterials possess distinctive features that enhance cellular attachment and multiplication along with differentiation capability and show promise for advancing bone regrowth therapies, the osseointegration of implants, and treating bone diseases.

### 4.2 Additional medical applications of nanotechnology

Researchers are applying nanotechnology more frequently for respiratory disease treatments through nanomaterials, demonstrating an exceptional ability to deliver drugs precisely to the lungs and create cutting-edge diagnostic devices for pulmonary conditions. Patients experience better results through enhanced treatment precision and effectiveness with this method, as detailed by Doroudian et al. (2021).

Medical professionals in ophthalmology are investigating how nanotechnology might offer treatments for conditions such as glaucoma. Nanomaterials provide improved drug delivery systems for ocular treatments while enabling novel surgical methods and developing advanced diagnostic tools for eye conditions. Recent innovations offer better management options for eye disease treatment, as supported by research findings (El Hoffy et al., 2023; Juliana et al., 2019).

Medical biophysics utilizes gold nanomaterials because their optical and electronic properties enable their use in imaging, therapeutic applications, and sensing. The unique properties of gold nanomaterials make them essential to improve diagnostic precision and therapeutic outcomes (Moore and Chow, 2021).

Theranostic nanomaterials transform medicine by combining therapeutic and diagnostic processes to enable simultaneous disease treatment and diagnosis. Due to this dual functionality, medical interventions become more efficient and effective (Rizzo et al., 2013).

The capabilities of MRI machines are improved through nanotechnology because they employ nanomaterials as contrast agents to enhance image quality. The enhancement enables the creation of new MRI-based diagnostic tools that offer clearer and more detailed images for improved diagnosis and monitoring (Sands and Levitin, 2004).

Nanotherapeutics for neurological diseases can penetrate the blood-brain barrier to transport the medication to the central nervous system. Targeted drug delivery through nanotherapeutics is critical in improving treatment results for Alzheimer’s and Parkinson’s diseases (Saxena et al., 2012).

Nanotechnology serves multiple functions in medical practice by improving imaging techniques and drug delivery systems to enhance diagnostic accuracy and treatment effectiveness. Nanomaterials enable the creation of new diagnostic tools and imaging agents that improve treatment precision and effectiveness for various diseases (Singh et al., 2020).

In cancer diagnostics and treatment, gold nanoparticles provide targeted drug delivery, photothermal therapy, and imaging applications. Applications demonstrate how gold nanoparticles perform effectively in cancer medicine (Singh et al., 2022).

Researchers are actively examining nanomedicines as potential glaucoma treatments through nanomaterials, demonstrating promise for ocular drug delivery and developing novel therapeutic approaches for the disease. Recent developments are being created to improve glaucoma treatment and management strategies (Zhai et al., 2021).

Through significant improvements in resolution, specificity, and sensitivity, imaging techniques benefit from the revolutionary effects of nanotechnology. Engineered nanomaterials serve as contrast agents that enhance the quality of tissue and organ images by producing clearer and more detailed visuals. The advancement offers improved capabilities to identify diseases at earlier stages and track treatment outcomes with greater precision (Butt and Bach, 2024). Nanotechnology-based molecular imaging agents enable the detection of cellular and molecular alterations, leading to enhanced diagnostic precision. Theranostics are multifunctional imaging agents that integrate diagnostic and therapeutic functions to deliver simultaneous treatment and imaging that enhance patient outcomes (Occhiutto et al., 2020). Magnetic resonance contrast enhancement for tumor visualization uses iron oxide nanoparticles to improve image clarity, which leads to more precise tumor detection and characterization (Na et al., 2009). For a detailed overview of various nanomaterial types, their medical applications, clinical statuses, key benefits, and references, please refer to Table 2.

**TABLE 2:** Nanomaterials with corresponding medical applications, clinical statuses, key benefits, and references.

Nanomaterial type	Medical application	Clinical status (Approved/Clinical trials)	Key benefits	References
Liposomes	Drug delivery for cancer	Approved	Reduced toxicity to healthy tissues	Chavda, 2019; Naseri et al., 2018
Magnetic nanomaterials	Hyperthermia therapy for cancer	Clinical Trials	Localized heat generation to kill cancer cells	Contera et al., 2020; Nel et al., 2023
Photosensitizing nanomaterials	Photodynamic therapy for cancer	Clinical Trials	Reactive oxygen species production to destroy cancer cells	Contera et al., 2020; Nel et al., 2023
Nanomaterial- based stents	Cardiovascular disease	Clinical Trials	Reduced restenosis and improved biocompatibility	Rizvi and Saleh (2018); Tekade et al., 2017; Occhiutto et al., 2020; Perera et al., 2023
Nanosensors	Early detection of heart attacks and other cardiovascular conditions	Clinical Trials	Biomarker detection for early diagnosis	Rizvi and Saleh (2018); Tekade et al., 2017; Occhiutto et al., 2020; Perera et al., 2023
Nanoantibiotics	Infectious diseases	Clinical Trials	Enhanced Delivery and efficacy of antibiotics	Chaturvedi et al., 2019; Pelaz et al., 2017; Chen et al., 2021
Antiviral nanomaterials	Infectious diseases	Clinical Trials	Inhibition of viral replication and enhanced immune responses	Chaturvedi et al., 2019; Pelaz et al., 2017; Chen et al., 2021
Nanomaterial- based vaccines	Infectious diseases	Approved	Improved vaccine efficacy and stability	Chaturvedi et al., 2019; Pelaz et al., 2017; Chen et al., 2021
Gold nanoparticles	Drug delivery across the blood- brain barrier for neurodegenerative diseases	Clinical trials	Targeted drug delivery to the brain	Chavda 2019; Khan et al., 2015; He et al., 2022
Nanocomposite scaffolds	Bone regeneration in orthopedics	Clinical Trials	Enhanced bone growth and repair	Chaturvedi et al., 2019; Tekade et al., 2017
Iron oxide nanoparticles	Contrast Agents in magnetic resonance imaging (MRI)	Approved	Enhanced visualization of tumors	Occhiutto et al. (2020)

### 4.3 Nanomaterial-based vaccines

Immunization has been revolutionized through the development of vaccines based on nanomaterial technology. Nanoparticles deliver antigens and adjuvants within these vaccines, strengthening the immune response while improving protection from infectious diseases. The primary nanoparticles used in vaccine development are liposomes, virus-like particles, and polymeric nanoparticles.

Nanomaterial-based vaccines deliver superior results to traditional vaccines because they enhance antigen presentation while creating powerful immune responses. COVID-19 mRNA vaccines from Pfizer-BioNTech and Moderna use lipid nanoparticles to transport mRNA, which directs cells to generate the viral spike protein, leading to an intense immune reaction.

Creating COVID-19 vaccines through mRNA technology and lipid nanoparticles represents a significant achievement. Clinical trials demonstrated that vaccines achieved high efficacy levels, which were critical in controlling viral transmission (Wilson and Geetha, 2022).

Nanomaterial-based vaccines provide multiple benefits compared to conventional vaccines. The application of nanoparticles improves antigen delivery and presentation, resulting in more robust and durable immune responses (Lozano et al., 2023). Nanotechnology enables faster vaccine production and development, evidenced by the swift creation of COVID-19 mRNA vaccines (Khurana et al., 2021). Nanoparticles improve vaccine stability and shelf life by safeguarding vaccine components against degradation (Shah et al., 2024). Scientists can design nanoparticles to interact with particular cells or tissues, which results in better vaccine performance (Alsaiari et al., 2024). The application of lipid nanoparticles in developing COVID-19 mRNA vaccines represents an exceptional success because these vaccines showed high effectiveness in trials and helped control viral transmission (Wilson and Geetha (2022).

## 5 Long-term effects of nanomaterials

The extended consequences of nanomaterials on human health and environmental systems remain an incomplete area of knowledge. Recent research has generated useful knowledge about possible risks and safety measures for nanomaterials (Schneider-Futschik and Reyes-Ortega, 2021).

### 5.1 Human health and environmental impact

Nanomaterials’ small size and high surface area give them unique properties that allow them to interact with biological systems in distinctive ways. Research findings show that particular nanomaterials trigger oxidative stress and inflammatory responses while causing toxic effects on cells (Hussain et al., 2024). Human cells show DNA damage and apoptosis when exposed to silver nanoparticles. Chronic exposure to nanomaterials has the potential to affect the immune system and cause lasting health problems.

The environmental effects of nanomaterials represent an important concern. The accumulation of nanomaterials in soil and water poses risks to ecosystems and biodiversity. Studies reveal that nanoparticles demonstrate toxicity to aquatic life, including fish and algae. The environmental pollution caused by nanomaterials’ manufacturing and disposal processes requires a thorough assessment of their full life cycle, from production through disposal.

### 5.2 Regulatory frameworks and future research

Frameworks for regulation are being established to guarantee the safe application of nanomaterials. The developed frameworks provide guidelines through which scientists can characterize nanomaterials, assess their safety, and test them in clinical settings. It is essential to develop standardized protocols to assess the long-term effects while mitigating potential risks (Tinkle et al., 2014).

Ongoing research remains essential for understanding the long-term effects of nanomaterials on biological systems and their environmental consequences. The testing process must comprehensively analyze the potential to trigger inflammation, oxidative damage to cells, DNA degradation, and additional harmful effects. It is crucial to assess the movement of nanomaterials through environmental systems and their impact on ecosystems and biodiversity.

It is essential to develop strategies that reduce potential risks. Safer-by-design approaches, surface modifications to reduce toxicity, and effective disposal techniques are included. Future research should focus on advancing targeted drug delivery systems and investigating nanotechnology applications for personalized medicine. Research should concentrate on advancing nanomaterials that can penetrate biological barriers to precisely release drugs at the target site and enable treatment customization to patients’ unique genetic profiles and clinical conditions.

The medical field can fully utilize the benefits of nanotechnology when it tackles these challenges through extensive research combined with appropriate regulatory protocols.

## 6 The role of artificial intelligence (AI) in nanomedicine and the development of personalized nanomedicines

The application of artificial intelligence (AI) in nanomedicine leads to revolutionary advances by creating more precise and personalized treatments while increasing efficiency. AI’s predictive and pattern-identification capabilities when processing large datasets significantly enhance the development and application of nanomaterials in medical fields.

### 6.1 AI in nanomedicine

The ability of AI to process massive and intricate datasets involving patient medical records and genomic information is revolutionizing nanomedicine (Holzinger et al., 2019; Adir et al., 2020; Sharma et al., 2024). AI can utilize patterns and trends found in data sets to create precise diagnostic tools and tailor treatment strategies (Zeb et al., 2024). AI systems can examine medical images to discover initial disease symptoms and predict how patients react to specific nanomedicine therapies (Holzinger et al., 2019; Sharma et al., 2024; Zeb et al., 2024).

AI-driven image analysis represents an effective approach to early cancer diagnosis. Medical image analysis using artificial intelligence detects early cancer signs more accurately through X-rays or magnetic resonance imaging than traditional diagnostic methods. Earlier diagnosis and treatment through this capability will improve patient outcomes (Oladele, 2024).

Personalized nanomedicine depends on genomic data analysis to detect specific mutations and biomarkers. The process enables predictions of individual patient responses to specific nanomedicines, which helps create customized treatment strategies. Patients receive nanomedicine treatments specifically adapted to their unique genetic makeup.

Diagnostic tools that utilize artificial intelligence can potentially transform disease management practices. These tools process medical records to detect patients at elevated risk for developing specific diseases. This identification process enables an early nanomedicine intervention that can stop disease progression or reduce its severity.

Artificial intelligence speeds up nanomedicine development and discovery through accurate predictions of how nanomaterials will interact with biological systems. The discovery of new therapeutic targets and optimization of drug formulations is achievable through multiple approaches (Agrahari et al., 2024). The analysis of extensive databases containing nanomaterial properties enables scientists to forecast interactions with biological systems, which helps identify nanomaterials suitable for drug delivery applications and imaging. Through AI-powered simulations, scientists can forecast how nanomedicines move through the body and how they are processed, which helps improve drug formulation and delivery methods, leading to better therapeutic results and improved safety. By tailoring them to fit patient-specific disease characteristics and genetic profiles, scientists develop nanomedicines with higher effectiveness and reduced side effects.

AI algorithms improve nanomedicine development by predicting nanomaterials’ physicochemical properties and biological interactions of nanomaterials that lead to superior efficacy and safety (Hosseinkhani et al., 2011). Prediction of nanomaterials’ size, shape, and surface properties for targeted applications such as drug delivery or imaging directs the synthesis process toward nanomaterials with specific desirable features. By predicting nanomaterial toxicity and immunogenicity, researchers can determine which nanomaterials are safer and more biocompatible for medical applications. The optimization of nanomaterials requires predictions of surface modifications that will improve drug-release characteristics and targeting capabilities.

AI supports clinical trial design and execution by selecting appropriate patient groups, forecasting treatment results, and continuously monitoring patient reactions in real-time (Agrahari et al., 2024). Implementing AI technology is expected to improve clinical trial operations by boosting their efficiency and effectiveness in nanomedicine. AI algorithms process patient data to determine which individuals would benefit the most from specific nanomedicines, thus increasing clinical trial efficiency. AI technology enables the prediction of the probability of success or failure of treatment, which supports patient stratification and treatment optimization. Real-time monitoring enables early identification of adverse effects during nanomedicine treatment, which allows healthcare professionals to adjust treatments promptly.

### 6.2 Development of personalized nanomedicines

Personalized nanomedicine customizes treatments to each patient’s genetic and clinical profile, leading to better treatment results and minimizing adverse effects (Holzinger et al., 2019). Combining AI technology with nanotechnology presents enormous potential for revolutionary medical advancements. The progression of AI technology will produce additional cutting-edge applications in nanomedicine that will improve healthcare effectiveness and personalization (Heydari et al., 2024).

We can discover mutations or biomarkers that make targets for nanomedicine treatment through AI analysis of genetic profiles. This capability enables the creation of personalized treatment plans that deliver better results while minimizing side effects (Holzinger et al., 2019; Sharma et al., 2024). AI enables clinicians to create nanomaterials that deliver drugs to targeted tissues or cells within the body, improving treatment precision and effectiveness for complex diseases such as cancer and neurodegenerative disorders (Fornaguera and García-Celma, 2017).

AI facilitates real-time observation of patient responses to nanomedicines, which helps clinicians modify treatment strategies as necessary. Treatment methodology enhances therapeutic results while minimizing adverse reactions (Hosseinkhani et al., 2011). Constant monitoring of patient response and adaptation of treatment strategy maintain therapeutic effectiveness and safety during treatment.

The development of nanorobots that can travel through human body systems to deliver medications and perform tissue repairs and disease marker detection is highly dependent on AI technology (Biswas, 2024). AI algorithms direct nanorobot movement and behavior, allowing these machines to execute complex bodily tasks independently (Biswas, 2024; Fatima et al., 2024). The autonomous capability enables accurate cellular interventions, which improves treatment outcomes while reducing unwanted side effects.

Personalized nanomedicine has reached new heights in healthcare care thanks to its integration with AI and nanotechnology. Personalized nanomedicine creates safer and more efficient therapies by customizing treatments based on each patient’s unique genetic and clinical characteristics. The expanding applications of artificial intelligence in nanomedicine will generate innovative solutions to complex medical challenges and enhance patient outcomes as research and technology advance.

## 7 Safety and ethical considerations and future scope and challenges of nanomedicine

### 7.1 Safety and ethical considerations

Important safety and ethical considerations emerge from the unique characteristics of nanomaterials, which include their minimal dimensions and extensive surface area. To achieve responsible advancement in nanomedicine, we must address these safety and ethical concerns (Juillerat-Jeanneret et al., 2015). Understanding the interaction of nanomaterials with biological systems requires thorough safety evaluations that explore their ability to cause oxidative stress, inflammation, and cytotoxicity (Singh et al., 2020). A combined approach using *in vitro* and *in vivo* studies remains crucial to assess nanomaterial biocompatibility and toxicity (Hussain et al., 2024).

The regulations continue to develop and change. The medical field needs standardized nanomaterial characterization and safety testing procedures to guarantee their effective and secure medical applications (Tinkle et al., 2014). Regulatory agencies must create detailed guidelines addressing the specific obstacles that nanomedicine presents.

The medical applications of nanotechnology present multiple ethical challenges that need a thorough evaluation. Patient consent, privacy protection, and unexpected results constitute the primary ethical considerations for nanotechnology in medicine (Malik et al., 2023). Patients must receive complete information on nanotechnology-based treatments while maintaining their privacy protection remains essential (Juillerat-Jeanneret et al., 2015). A development of ethical frameworks is needed to manage nanotechnology’s broader social implications, including access to nanomedicine, environmental impacts, and risks of misuse or unintended outcomes (Dave et al., 2021).

The advancement in nanotechnology might allow us to use nanomaterials to enhance human abilities beyond typical limits. Using nanotechnology to enhance human capabilities beyond normal limits introduces ethical dilemmas regarding what constitutes “normal” while creating a possible divide between enhanced and nonenhanced individuals. No one knows the long-term effects of these enhancements, which creates concern about potential dangers to both people and communities. Ethical guidelines and regulations for nanotechnology applications in medicine must be developed to guarantee responsible and beneficial use of this technology (Dave et al., 2021).

The increasing integration of nanomaterials into medical applications prompts questions about their environmental consequences. The complete life cycle of nanomaterials requires careful examination from their production to disposal stages, while sustainable manufacturing and waste management practices must be developed to reduce harmful environmental impacts (Hussain et al., 2024).

### 7.2 Future scope and challenges

The future holds great potential across diagnostic and treatments along with regenerative medical practices. The full potential of nanotechnology in medical applications will only be reached by solving multiple existing challenges.

Research analyzes challenges and opportunities for moving nanotechnology from laboratory research to clinical applications while focusing on safety, efficacy, and regulatory obstacles (Min et al., 2015). The path to clinical translation of nanomedicine requires further research and development efforts to address existing barriers (Sindhwani and Chan, 2021). The analysis of obstacles and methods to bring nanotechnology to market highlights the need to meet market demands while ensuring regulatory compliance and protecting intellectual property (Shapira and Wang, 2009).

A priority for future research should be to create complex nanomaterials that can perform disease diagnosis and treatment while monitoring patient health at the same time. The popularity of theranostic nanomaterials that integrate therapeutic and diagnostic capabilities will increase as they deliver more personalized and precise medical treatments (Contera et al., 2020). The fusion of nanotechnology with fields such as artificial intelligence and biotechnology will facilitate the development of smart nanomaterials that react to specific stimuli and adjust to physiological conditions, improving their performance and safety (Adir et al., 2020).

Understanding the intricate interactions between nanomaterials and biological systems remains essential for advancing research. Research efforts should focus on the protein corona surrounding nanomaterials, how cells absorb these particles, and their prolonged impacts within biological systems (Fadeel et al., 2013). The production of nanomaterials involves intricate procedures that demand high expenditures. The development of scalable and cost-effective production methods remains essential to achieve widespread adoption of nanomedicine (Gaspar, 2010).

The development of nanomedicine requires comprehensive regulatory frameworks and ethical guidelines to maintain safety and responsibility. Resolution of patient consent, privacy, and environmental impact remains essential (Juillerat-Jeanneret et al., 2015). Through tackling these obstacles and maintaining innovative efforts, the nanomedicine sector will make great strides in delivering modern treatments for critical health problems.

## 8 Conclusion

This review has thoroughly examined the transformative impact of nanotechnology on healthcare, focusing on its applications in imaging, diagnosis, drug delivery, and regenerative medicine. Key findings highlight the unique structural characteristics, synthesis methods, and functionalization of nanomaterials, which significantly enhance their effectiveness in medical applications. The review underscores the substantial advantages of targeted drug delivery systems, including using nanomaterials as contrast agents in imaging and developing *in situ* diagnostic tools. Furthermore, the potential of nanotechnology in treating cancer and cardiovascular disease was discussed, emphasizing its pivotal role in improving therapeutic outcomes and patient care.

Nanotechnology has immense potential to revolutionize medicine by enabling more precise, efficient, and personalized treatments. Its ability to target specific cells and tissues, enhance imaging techniques, and provide multifunctional capabilities positions nanotechnology as a critical tool in modern healthcare. Integrating nanotechnology with other emerging fields, such as artificial intelligence and biotechnology, further expands its potential, paving the way for innovative solutions to complex medical challenges.

Continued research and development are essential to fully realizing the medical benefits. Future research should focus on the development of multifunctional nanomaterials, the understanding of their biocompatibility and toxicology, and the exploration of their applications in personalized medicine. Addressing regulatory, safety, and ethical challenges is crucial to ensure safe and effective use in clinical settings. By pursuing these research directions and fostering interdisciplinary collaboration, the medical community can harness the full potential of nanotechnology to improve patient outcomes and advance healthcare.
